# Design and control of a novel pneumatic soft robot based on the improved particle swarm optimization algorithm

**DOI:** 10.1371/journal.pone.0333187

**Published:** 2025-09-25

**Authors:** Hongjun Meng, Changbao Zhou, Xingbo Yang, Peiqi Zhao, Wei Zhang

**Affiliations:** School of Automation and Software, Shanxi University, Taiyuan, China; Southeast University, CHINA

## Abstract

Inspired by the flexible structures found in soft organisms in nature, researchers have developed a variety of novel soft robots using flexible materials. Compared to traditional rigid robots, soft robots offer advantages such as lighter weight, greater flexibility, higher degrees of freedom, and improved safety in human-robot interaction. However, designing and controlling soft robots remains a significant challenge. This paper proposed a novel design approach for a bio-inspired quadruped soft robot. Firstly, a hexagonal mesh structure for a quadruped soft robot was proposed, and the pneumatic actuator suitable for the soft structure was designed to enable the robot’s leg movements, such as extension and bending. The body and overall structure of the robot were also systematically designed. Furthermore, a data-driven modeling method for the soft actuator was introduced, alongside an Improved Particle Swarm Optimization algorithm for fine-tuning PID control parameters. Finally, the prototype of the quadruped soft robot was constructed, and the control system was implemented. The proposed soft actuator model was validated, and the effectiveness of the proposed optimized algorithm was evaluated. Experimental results demonstrated that the application of the soft control model and the control parameter optimization algorithm reduced tracking angle errors by more than 50%, resulting in improved control accuracy and greater stability.

## 1. Introduction

Robots are now widely utilized in industries such as healthcare, automotive, aerospace, food, and agricultural production, significantly boosting productivity across these sectors [1,2]. However, most robots still rely on rigid structures, which pose challenges such as excessive weight, limited interactivity, and poor adaptability to various environments. Soft robots [3], representing an emerging form of robotics, have garnered increasing attention. These robots are often inspired by soft-bodied organisms, such as octopuses, snakes, and worms. Their flexible bodies allow them to interact with various objects safely which does not pose the risk of damaging other objects. In contrast to rigid robots that have a limited number of joints and degrees of freedom [4], soft robots are typically constructed from materials like silicone, using techniques such as bonding, molding, or 3D printing [5]. This construction grants soft robots exceptional flexibility and safety, making them highly promising in fields such as healthcare, services, and rescue. Soft robotics has become a key area in the development of robotic technology [6]. There are various actuation methods for soft robots, including electro-active polymer actuation [7–11], electromagnetic actuation, combustion-based actuation and pneumatic actuation. Among these, pneumatic actuation plays a significant role in the actuation of soft robots due to its advantages of lightweight design, high efficiency, environmental friendliness, and strong adaptability to different environments.

Rafsanjani designed a pneumatic snake-like robot drew inspiration from the art of kerygma [12]. When the silicone rubber tube is inflated, scales on the robot’s surface protrude and anchor onto the contact surface, propelling the robot forward. Researchers from the University of Southern Denmark developed a soft actuator modeled after the interwoven structure of worm muscle fibers [13], which is applicable for gastrointestinal endoscopy or sewer inspections. Robert developed a humanoid underwater robot driven by electromagnetic actuation [14], capable of autonomously detecting, tracking, and collecting debris from the seafloor. Researchers from the University of Iowa proposed a muscle actuator inspired by the muscle systems of octopuses [15], which is actuated by an artificial muscle known as twisted and coiled actuators.

In recent years, the control technologies for soft robots have become increasingly diverse. Among them, modeling approaches are typically divided into two categories: model-based control and model-free control [16–19]. Model-based control techniques include methods such as piecewise constant curvature, Caserta rod theory, and finite element analysis. Model-free control techniques, such as neural networks and deep reinforcement learning, have been applied to control soft robots. Control algorithms are also one of the core aspects of robotic control technology. Many researchers have investigated the characteristics of both open-loop and closed-loop control strategies for soft robots using various control algorithms. Among them, closed-loop control employs both external and embedded sensors, resulting in higher control precision. Current control algorithms for soft robots can be broadly categorized into classical control and intelligent control. Classical control includes approaches such as PID control, adaptive control, and disturbance compensation control [20–22], while intelligent control encompasses data-driven methods and bio-inspired approaches. Cao [23] developed a data-driven model for the soft crawling robot. Robinson [24] proposed a control strategy for a soft robotic arm using sliding mode control combined with neural networks. Zhu [25] introduced a control strategy for a soft-swallowing robot based on the central-pattern- generator. By applying multiple control algorithms, the control of soft robots has become more precise.

In summary, researchers have made improved advances in various aspects of soft robotics. Nevertheless, due to the high flexibility and degrees of freedom inherent to soft robots, their control remains a substantial challenge, and their structure design and manufacturing still involve considerable complexity. The quadruped robot, a well-established robotic form, offers notable stability and mobility, making it suitable for navigating diverse terrains [26]. By incorporating the highly flexible and deformable soft materials into the structural design of quadruped robots, it becomes possible to more accurately mimic the movement of quadrupedal organisms and enhance the robot’s adaptability and stability in irregular environments. This paper presents a novel pneumatically actuated quadruped soft robot, thoroughly investigating its structural design and control mechanisms. Specifically, this paper proposes an Improved Particle Swarm Optimization algorithm for the automatic tuning of PID controller parameters. Finally, the designed robot successfully demonstrates walking capabilities.

## 2. Design of the soft quadruped robot

### 2.1 Structure and actuator design for the soft leg

**Fig 1:**
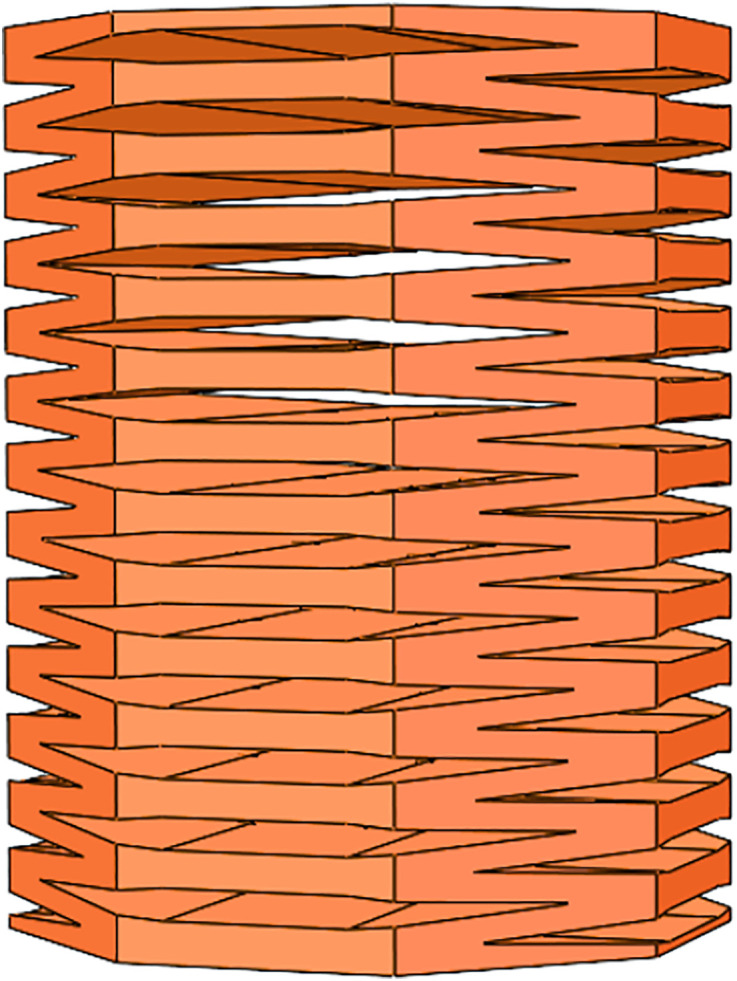
Main structure of the octagonal prism.

**Fig 2:**
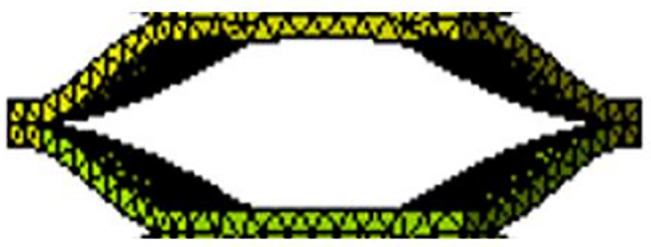
The structure of a single chamber.

**Fig 3:**
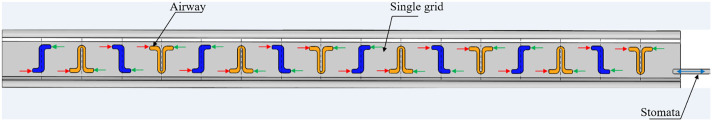
The structure of the pneumatic actuator.

**Fig 4:**
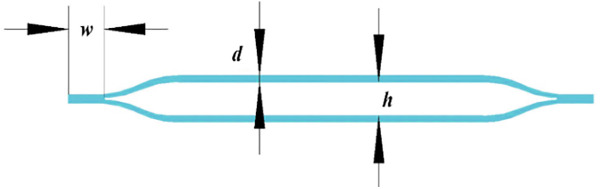
Side view of a single air pouch.

Previously, I published an article on a quadruped soft robot controlled by a PID optimized with a genetic algorithm [27]. This paper proposes a novel soft leg structure together with a control algorithm specifically designed for the robot. The leg is constructed in the form of a regular octagonal prism, which combines the flexibility of cylindrical legs—allowing movement in all directions—with the stability of prismatic legs.The main body structure and the single-chamber structure are shown in Figs 1 and 2, respectively. Considering the influence of the wall thickness of a single hexagonal chamber on structural deformation, finite element analysis was conducted on a single chamber and the structure was tested with different wall thicknesses. When the wall thickness is 4 mm, the deformation size is applicable. The pneumatic actuator internally utilizes smaller tubes of different specifications to partition the air chambers. The long strip-shaped pneumatic driver is divided into sixteen uniformly sized gas bags installed in eight hexagonal chambers using folding and embedding methods, each containing two gas bag units. When installing the actuator into the hexagonal mesh structure, the mesh structure will deform as the actuator gradually expands. One chamber has two air pockets, allowing the soft mechanical leg to achieve significant elongation or bending movements. The structure of the pneumatic actuator is shown in Fig 3. Each small airbag is an equally divided square, and a small air guide hose with alternating long and short lengths is used. The short tube allows gas to flow between adjacent groups of airbags, ensuring that the contraction of a single chamber is not affected, and the long tube is positioned between two adjacent hexagonal chambers, which ensures that the gas flow between the two adjacent chambers is not affected. When inflating the actuator, the air pressure acts on the inner wall of the actuator, causing a single airbag shown in Fig 4 to expand, resulting in a continuous increase in airbag height *h*. The deformation of the airbag causes the driver to generate outward expansion force. During the deflation operation, each airbag returns to its original state. The edge width *w* and wall thickness *d* remain unchanged during the above inflation and deflation process, ensuring the stability of a single airbag structure.

**Fig 5:**
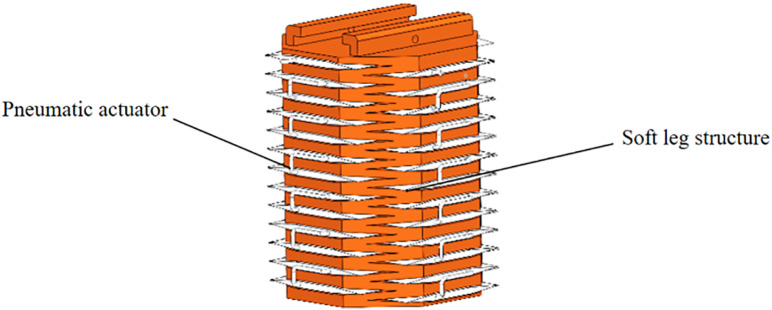
The structure of the single soft robotic leg.

**Fig 6:**
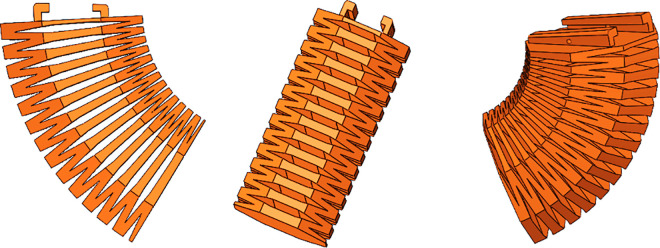
Unilateral bending, elongation, and diagonal bending of the soft leg.

By uniformly integrating pneumatic actuators into the hexagonal structure of the leg, the soft robotic leg shown in Fig 5 is constructed. The designed soft leg with the hexagonal mesh structure can achieve unilateral bending, elongation, and diagonal bending as shown in Fig 6. when each chamber is subjected to outward expansion force. To enable the soft robot to achieve the above deformation and walk forward, four pneumatic drivers are folded and installed in the hexagonal chambers in the soft leg. The deformation of the soft leg is achieved by inflating the pneumatic drivers, and the leg structure is restored to its original state by deflation. Thus, the designed structure combined with the pneumatic driver can be used to complete the deformation of the soft leg.

### 2.2 Overall structure of quadruped robot

**Fig 7:**
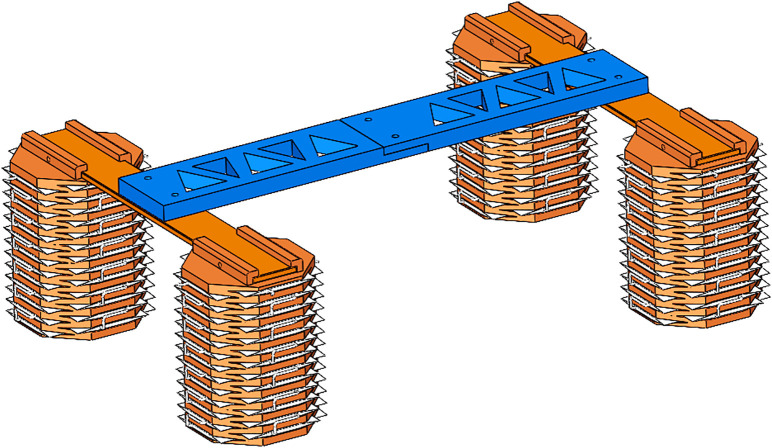
The overall structure of the soft quadruped robot.

The horizontal frame present on both sides of the torso is designed as a width-equal structure, ensuring symmetry and stability throughout the robot’s midsection. The vertical bracket is specifically designed with a hollow triangular structure, which serves the critical function of reducing the overall weight of the torso. This reduction in mass is advantageous as it directly lessens the load exerted on the robot’s legs, thereby enhancing its mobility and efficiency. The complete structural layout of the soft quadruped robot is illustrated in Fig 7. The dimensions of the robot are carefully defined to achieve optimal functionality: the total length of the soft quadruped robot measures 330 mm, while its width extends to 250 mm, and its height reaches 110 mm. These proportions are chosen to balance the robot’s structural integrity with its intended movement capabilities.

### 2.3 Kinematic analysis of the quadruped soft robot

In traditional rigid robots, the Denavit–Hartenberg (D-H) method is commonly used to describe kinematics. This method analyzes the robot’s motion by establishing the relationship between the pose of the end-effector of a rigid robotic leg or arm and the variables of each joint. However, the robot studied in this paper is a soft robot whose leg structure is made of flexible TPU(Thermoplastic Polyurethane) material via 3D printing. Unlike rigid robots, the legs of soft robots do not have clearly defined joint structures, and thus lack parameters such as rigid joint bending angles. Therefore, an equivalent D-H method is introduced to enable the kinematic analysis.

**Fig 8:**
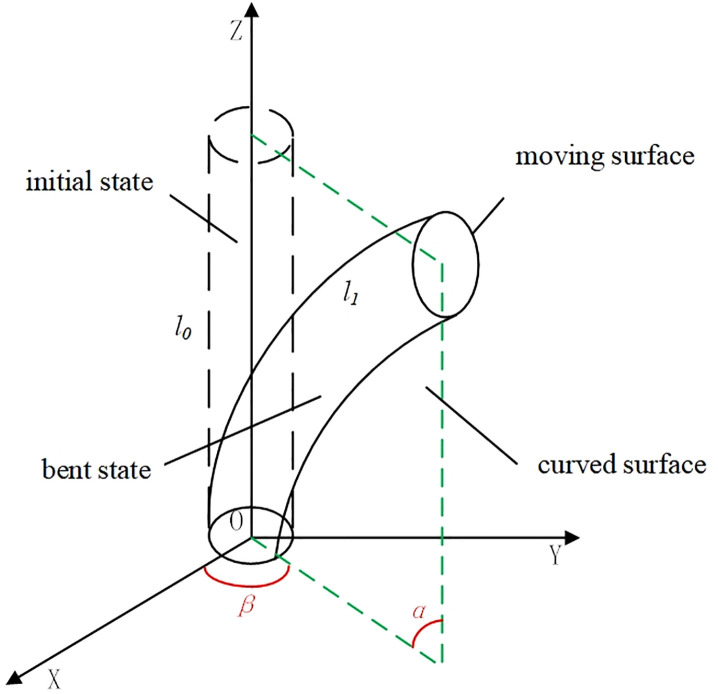
Schematic diagram of soft leg motion.

The soft robotic leg is modeled as a cylindrical segment. A spatial coordinate system is established at the center of the plane on the side with the connecting device, following the right-hand rule, as shown in Fig 8.

To analyze the bending state, the plane in which the cylindrical segment bends is referred to as the *bending plane*, which is indicated by the green dashed frame in the Fig 8.The angle between the fixed plane and the moving plane is defined as the *bending angle* α , and the angle between the bending plane and the XOZ plane is referred to as the *rotation angle* β,the initial length of the cylindrical segment is denoted as $l_{0}$, after bending, the length of the bending arc is $l_{1}$, the length at a certain moment during bending is $l_{t}$, the radius of the bending arc is r, and the coordinates of the center point of the moving plane in space are denoted as $O_{1}\left(x,y,z\right)$, based on this, the following equations are obtained.


$$\text{r}=\frac{{\text{l}}_{\text{t}}}{\alpha }$$ (1.1)

$$O_{\text{1}}=(\begin{array}{c} x\\ y\\ z \end{array})=(\begin{array}{c} (\text{1}-cos\alpha )\cdot cos\beta \\ (\text{1}-cos\alpha )\cdot sin\beta \\ sin\alpha \end{array})\cdot \text{r}$$ (1.2)

Under the constant curvature model, the pose of the moving plane in space can be determined. By translating the center point O of the fixed plane to the center point $O_{\text{1}}$ of the moving plane, the corresponding translation matrix is given as follows:


$$T_{1}=\left(\begin{array}{cccc} {}*20c1 & 0 & 0 & (1-\cos\alpha )\cdot \cos\beta \cdot r\\ 0 & 1 & 0 & (1-\cos\alpha )\cdot \sin\beta \cdot r\\ 0 & 0 & 1 & \sin\alpha \cdot r\\ 0 & 0 & 0 & 1 \end{array}\right)$$ (1.3)

By rotating the coordinate system around the Z-axis by an angle β, the resulting rotation matrix $R_{\text{1}}$ is given by:


$$R_{1}=Rot\;(Z,\;\beta )=\left(\begin{array}{cccc} {}*20c\cos\beta & -\sin\beta & 0 & 0\\ \sin\beta & \cos\beta & 0 & 0\\ 0 & 0 & 1 & 0\\ 0 & 0 & 0 & 1 \end{array}\right)$$ (1.4)

By rotating the coordinate system around the Y-axis by an angle α, the resulting rotation matrix $R_{2}$ is given by:


$$R_{2}=Rot\;(Y,\;\alpha )=\left(\begin{array}{cccc} {}*20c\cos\alpha & 0 & \sin\alpha & 0\\ 0 & 1 & 0 & 0\\ -\sin\alpha & 0 & \cos\alpha & 0\\ 0 & 0 & 0 & 1 \end{array}\right)$$ (1.5)

By rotating the coordinate system around the Z-axis by an angle -β, the resulting rotation matrix $R_{\text{3}}$ is given by:


$$R_{3}=Rot\;(Y,\;\alpha )=\left(\begin{array}{cccc} {}*20c\cos\beta & \sin\beta & 0 & 0\\ -\sin\beta & \cos\beta & 0 & 0\\ 0 & 0 & 1 & 0\\ 0 & 0 & 0 & 1 \end{array}\right)$$ (1.6)

Thus, the homogeneous transformation matrix T describing the transformation of the coordinate system from the fixed plane to the moving plane can be obtained as:


$$T=T_{1}R_{1}R_{2}R_{3}=\left(\begin{array}{cccc} \cos^{2}\beta \cos\alpha +\sin^{2}\beta & \cos\beta \cos\alpha \sin\beta -\cos\beta \sin\beta & \cos\beta \sin\alpha & (1-\cos\alpha )\cos\beta \cdot r\\ \cos\beta \cos\alpha \sin\beta -\cos\beta \sin\beta & \sin^{2}\beta \cos\alpha +\cos^{2}\beta & \sin\beta \sin\alpha & (1-\cos\alpha )\sin\beta \cdot r\\ -\cos\beta \sin\alpha & -\sin\beta \sin\alpha & \cos\alpha & \sin\alpha \cdot r\\ 0 & 0 & 0 & 1 \end{array}\right)$$ (1.7)

**Fig 9:**
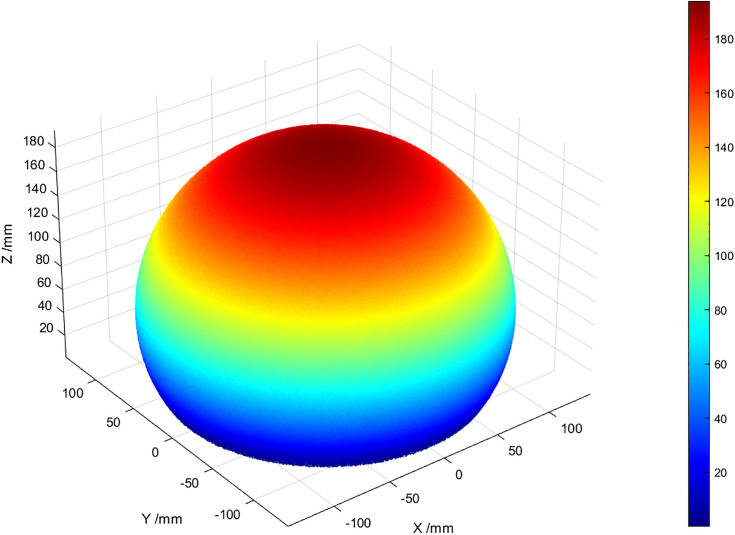
Three-dimensional workspace of the foot-end.

Based on the single-leg forward kinematics expression (1.7) and the variations of the virtual joint variables, the end position of each soft leg can be determined. Experimental measurements show that the cylindrical segment length varies between 110 mm and 194 mm.Since the virtual joint variables of the soft legs can be randomly generated in each movement, a scientifically grounded statistical model is established to characterize the end-effector positions.This method transforms the complex research object and computational problem into the simulation and calculation of random variables and their statistical properties, thereby simplifying the research and reducing computational complexity.The statistical modeling and computations were implemented in MATLAB, resulting in the workspace of the foot-end. The 3D workspace of the foot-end is shown in Fig 9.

## 3. Establishment of control model for soft quadruped robot

The control model of the soft structure is established based on a data-driven method. The locomotion of the robot mainly depends on the bending of the soft legs, which is achieved by controlling the amount of gas entering the pneumatic actuators. Therefore, the bending degree of the soft leg is a key parameter. Experimental measurements were then carried out.

**Fig 10:**
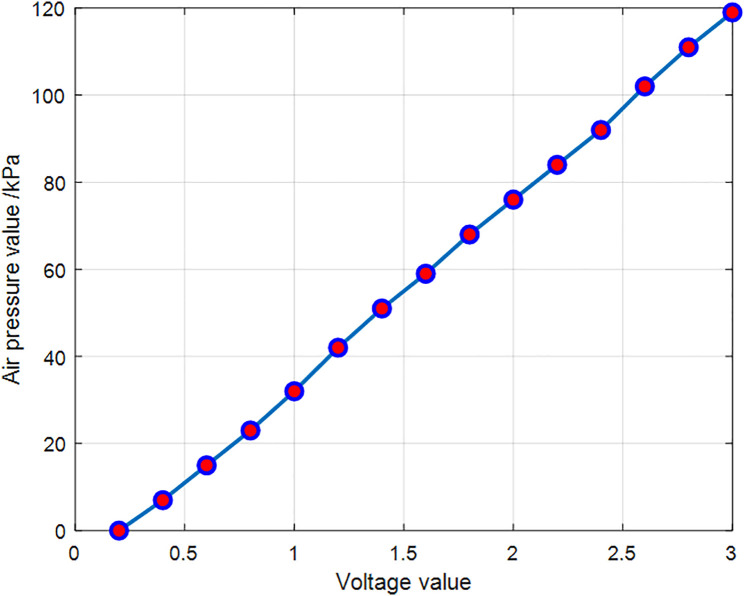
Proportional valve inputs and outputs.

Before the experiment, the input–output characteristics of the electro-pneumatic proportional valve were calibrated, and the relationship between the input signal and the output pressure was obtained, as shown in Fig 10. The results indicate that the input voltage signal is proportional to the output pressure, which demonstrates that the internal pressure of the pneumatic actuator increases uniformly during inflation. The gas supply is stable and does not adversely affect the experiment. Thus, in the experimental process, the voltage signal of the proportional valve can be directly used to represent the output pressure.

**Fig 11:**
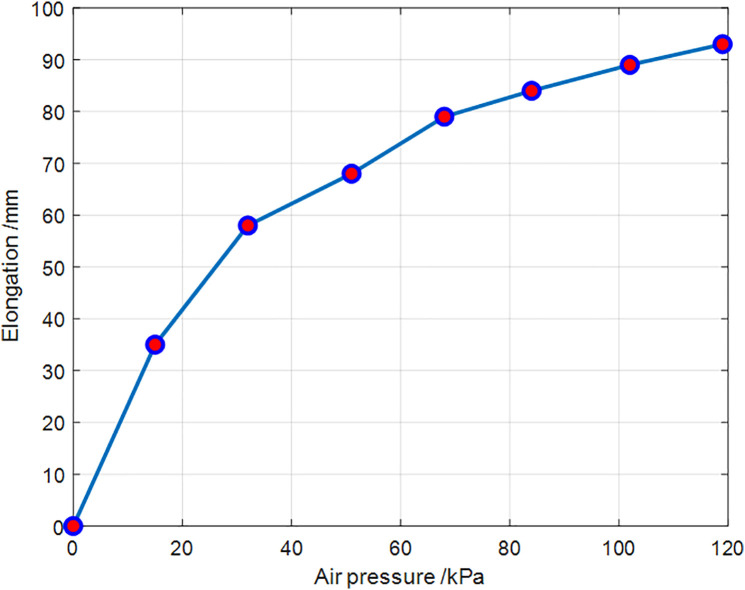
Air pressure versus elongation.

At the beginning of inflation, the total length of the soft leg with the actuator installed was measured multiple times, and the average value was taken to reduce measurement error. The fixed actuator was then inflated by issuing commands from the charging program to the PLC(Programmable Logic Controller), allowing the pressure to vary from low to high. At each pressure point, the length of the soft leg was recorded three times, and the average was calculated. After measurement, the elongation at each pressure point was obtained by subtracting the initial leg length from the measured length. The results were summarized and plotted to obtain the relationship between elongation and pressure, as shown in Fig 11.

**Fig 12:**
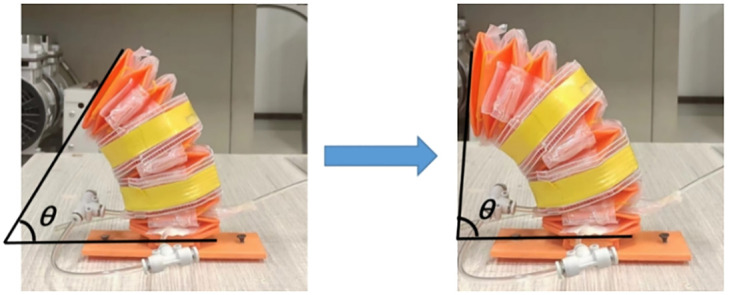
Soft leg bending angle.

**Fig 13:**
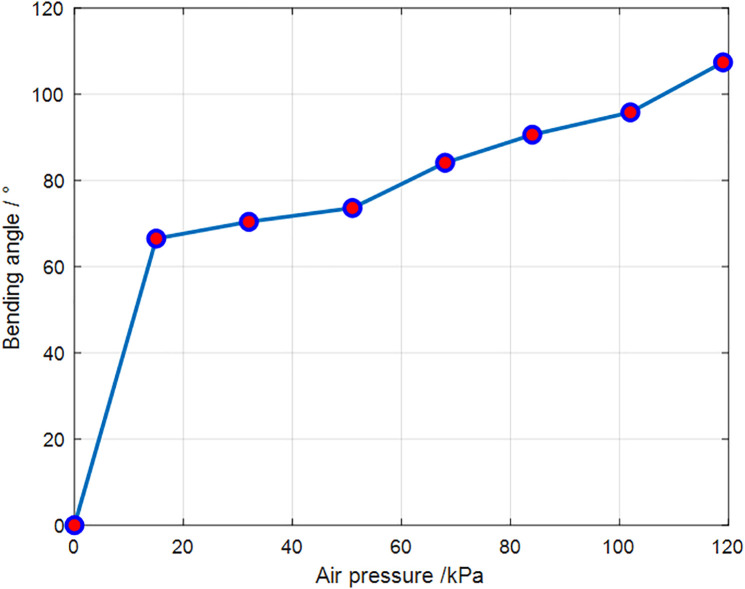
Air pressure versus bending angle.

Subsequently, the bending angle was measured. One side of the soft leg was randomly selected for inflation. To reduce error during angle measurement, multiple measurements were taken and averaged. Fig 12 illustrates the bending of the soft leg at a specific moment. The angle θ marked in the figure is the bending angle measured in this study. As the pressure increases, the bending angle also increases until the maximum deformation of the structure is reached. The recorded angle data were processed and plotted, and the resulting curve is shown in Fig 13. It can be seen that when the pressure is zero, the bending angle is also zero. At 17 kPa, the soft leg structure already exhibits significant deformation, indicating high flexibility and fast response to small inputs. As the pressure continues to increase, the bending angle grows until the structure can no longer deform. Considering the influence of measurement error, the relationship between pressure and bending angle within this range can be approximated as linear.

**Fig 14:**
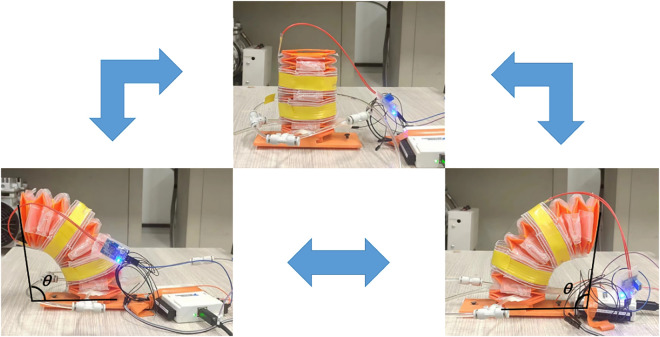
Data collection for developing the control model for the soft leg.


The state of the leg structure bending clockwise or counterclockwise at a certain input value is shown in Fig 14. Within a specified period, each input air pressure of the pneumatic actuator is set, while the bending angle of the soft leg is recorded simultaneously. Taking the valve voltage as an input signal, the system measures the bending angle as the valve voltage varies. The spatial state equation is expressed in Eq. (1.8), and the corresponding transfer function is Eq. (1.9).

$$\{\;\;\;\begin{array}{c} {}*20cdx/dt=Ax(t)+Bu(t)+Ke(t)\\ y(t)=Cx(t)+Du(t)+e(t) \end{array}$$ (1.8)

$$G(s)=C({sI-A)}^{-1}B+D$$ (1.9)

The construction of the data-driven control model for the soft structure involves three primary steps. First, data preprocessing is performed to filter, resample, or transform the raw data, thus creating a new dataset based on the original data. Next, the model is estimated using the preprocessed data based on Eq. (1.8). This involves selecting a model that provides the best fit for the measured data. The final step is to analyze the model by calculating the goodness-of-fit, which reflects the identification accuracy. A higher goodness-of-fit indicates higher precision of the control model. Therefore, a suitable state-space equation can be established based on this parameter, which can be used for subsequent control of soft legs.

## 4. PID tuning based on improved particle swarm optimization

### 4.1 PID control

PID control is widely adopted in various industries due to its advantages of simple composition, strong adaptability, strong robustness, and easy maintenance. The input-output expression of the PID controller is:


$$u\left(t\right)=K_{p}\left[e\left(t\right)+\frac{\text{1}}{T_{i}}\int_{0}^{t}e\left(t\right)dt+T_{d}\frac{de\left(t\right)}{dt}\right]$$ (1.10)

$K_{p}$is the proportional coefficient, *T*_*i*_ is the integral time constant, *T*_*d*_ is the derivative time constant, *e(t)* is the system error, and *u(t)* is the output variable of the controller. The integral coefficient $K_{i}$ and differential coefficient $K_{d}$ are respectively represented as:


$$K_{i}=\frac{K_{p}}{T_{i}}$$ (1.11)

$$K_{d}=K_{p}T_{d}$$ (1.12)

### 4.2 GA-PID control

Genetic Algorithm (GA) is an intelligent optimization algorithm that simulates the process of biological evolution. It is widely used in search and optimization problems.The algorithm primarily operates through chromosome encoding, evaluation of the fitness function for each individual, and genetic operations. Each individual represents a potential solution to the problem.By calculating fitness values and performing genetic operations such as selection, crossover, and mutation, the population evolves generation by generation toward the optimal solution.Through successive iterations of survival of the fittest, the population continues to evolve and eventually converges to the optimal solution of the given problem.

First, it is necessary to set the initial population size, which corresponds to the number of $K_{p}$, $K_{i}$, and $K_{d}$ parameter combinations, and define the parameter ranges to ensure more accurate identification of suitable values.Next, an appropriate fitness function must be determined, taking into account the potential impact of disturbances on the performance of the control system.Finally, the optimal PID parameters are obtained through the genetic operations of selection, crossover, and mutation.

### 4.3 Improving particle swarm optimization algorithm to optimize PID control

Particle Swarm Optimization (PSO) is an intelligent optimization algorithm with advantages such as strong adaptability and fast convergence, making it suitable for parameter tuning in PID controllers. PSO was first proposed by Kennedy and Eberhart in 1995 [28]. Due to its simple design and fast convergence, it has become one of the most popular evolutionary algorithms. The search direction is adjusted based on the optimal position and historical flight experience. However, due to the algorithm’s randomness and local search characteristics, it is prone to falling into local optima. Researchers have proposed an adaptive inertia weight method [29] that automatically adjusts the weight coefficients to avoid local optimum traps. In addition, the initialization of the algorithm has a significant impact on the search process. This paper proposes an Improved Particle Swarm Optimization (IPSO) method by combining a multi-start strategy with adaptive inertia weights, aiming to further prevent the algorithm from being trapped in local optima.

The PSO algorithm can be represented as follows: in a search space of dimension *N*, with *n* particles, the spatial position of the *i*-th particle is defined as:


$$X_{i}=\left(x_{i1},x_{i2},\cdots ,x_{iN}\right)$$ (1.13)

The velocity of the particle is defined as *V*_*i*_, the personal best position *P*_*i*_ refers to the optimal position found by the particle at the current moment, and the global best position *P*_*g*_ refers to the optimal position found by the entire particle swarm. Particles from different starting points continuously update their velocities according to the following equation:


$$V_{i}^{t+1}=\omega \cdot V_{i}^{t}+c_{1}\cdot r_{1}\cdot \left(P_{i}^{t}-X_{i}^{t}\right)+c_{2}\cdot r_{2}\cdot \left(P_{g}^{t}-X_{i}^{t}\right)$$ (1.14)

ω represents the particle’s inertia weight, *c*_1_,*c*_2_,*r*_1_,*r*_2_ are constant coefficients.

Meanwhile, the particles at each starting point are also constantly updating their positions in the current space, based on the following equation:


$$X_{i}^{t+1}=V_{i}^{t+1}+X_{i}^{t}$$ (1.15)

Here, *X*_*i*_^*t+1*^, *X*_*i*_^*t*^ represents the positions of particle *i* at times *t* and *t* + 1, respectively.

The multi-start strategy involves using multiple different starting points to initialize the optimization algorithm, thereby increasing the diversity of the search process and adapting to different search spaces to enhance the robustness. Moreover, this strategy enables parallel execution of the algorithm, which improves its running speed. Besides, inertia weight is a critical parameter that balances global and local search; a larger weight favors global search, while a smaller weight favors local search. This paper employs a logarithmic decay method to adjust the inertia weight using the logarithmic function. This method allows for quickly finding the global optimum in the early stages and stabilizing the search in the later stages, which helps obtain better local optimum solutions.

The range of the inertia weight is defined, and it undergoes logarithmic decay within this range, achieving adaptive variation of the inertia weight. The expression for logarithmic decay is as follows:


$$\omega =\omega_{\min}+\left(\omega_{\max}-\omega_{\min}\right)\times \frac{\log\left(I_{\max}-I+1\right)}{\log\left(I_{\max}\right)}$$ (1.16)

*ω*_*max*_ represents the maximum value of the inertia weight, *ω*_*min*_ represents the minimum value of the inertia weight, *I*_*max*_ is the maximum number of iterations, and *I* is the current iteration number. This ensures that the inertia weight decays logarithmically from the set maximum value to the minimum value, thereby achieving adaptive variation of the inertia weight. The performance of the PID controller is determined by three parameters: $K_{p}$,$K_{i}$,and $K_{d}$. Thus, the optimization of the PID controller parameters becomes a three-dimensional optimization problem. To summarize, the process of PID control optimization based on the improved PSO algorithm is as follows:

1. Define the number of parameters to be optimized is 3, the number of particles is set to 50, the number of starting points is 3, and the maximum number of iterations is set to 50.
2. Randomly initialize the positions and velocities of particles at multiple starting points.
3. Use the integral of Time-weighted Absolute Error(ITAE) index as the fitness function to evaluate the fitness value of each particle. The fitness function is defined as:

$$J_{i}=\int_{0}^{\infty }t\left|e\left(t\right)\right|dt$$ (1.17)

where *t* represents time, and *e(t)* denotes the difference between the desired output and the actual output at time *t.* This index accumulates errors over a period of time, and the smaller the value, the better the system’s control performance.

1. Update and adjust the velocity *V*_*i*_ and position *P*_*i*_ of each particle.
2. Recalculate and compare the fitness value *J*_*i*_ of each particle.
3. Update the velocity and position of the particles continuously to obtain the global best particle *X*_*i*_.
4. If the maximum number of iterations is reached, the loop ends; otherwise, return to Step 3.

**Fig 15:**
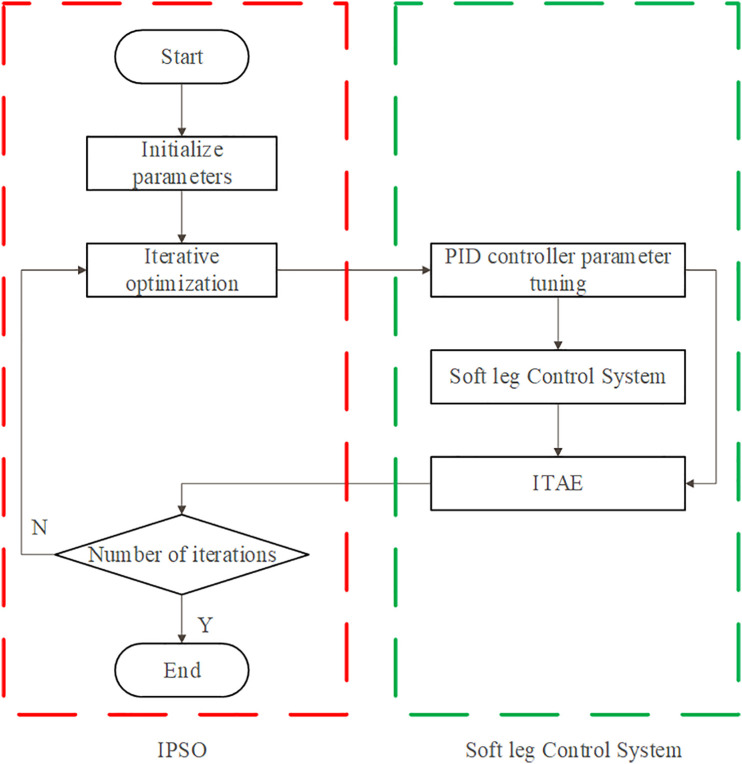
The flowchart of the PID control based on the IPSO algorithm.

The flowchart of the PID control based on the IPSO algorithm is depicted in Fig 15 The iterative optimization process includes several key steps: logarithmic decay of the inertia weight, updating of particle velocity and position, calculation of the fitness value, and accumulation of the iteration count. Once these iterative steps are completed, the optimized parameter values are derived, enabling the implementation of PID control for the soft leg.

### 4.4 Simulation

**Fig 16:**
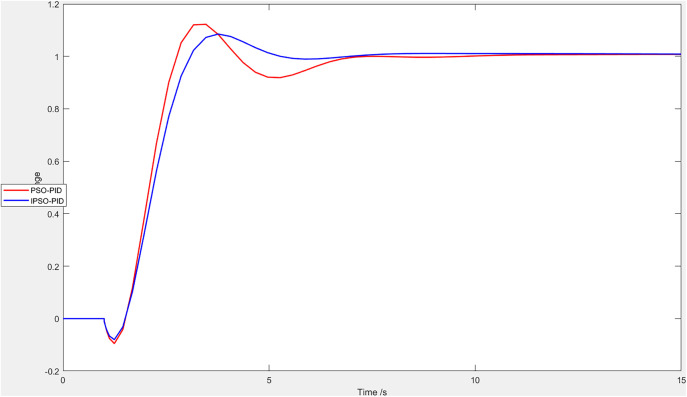
comparison of the PID control performance using the IPSO and PSO algorithms.

Set the input signal as the step signal to compare the PID control performance using the IPSO and PSO algorithms. As shown in Fig 16, the control system with the improved algorithm exhibits a smaller overshoot and a shorter settling time. Therefore, the IPSO algorithm allows the system to reach a stable state more quickly.This demonstrates that the IPSO algorithm achieves better performance in the soft leg control of the quadruped robot.

**Fig 17:**
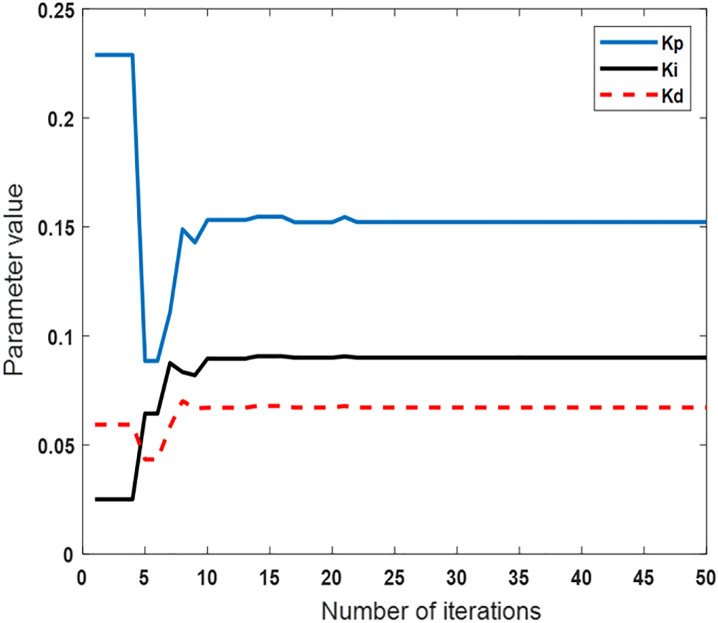
PID controller parameters optimization process.

The PID parameter optimization process after applying the IPSO algorithm is illustrated in Fig 17. The variation trends of the three PID controller parameters are visually displayed as the number of iterations increases. The iteration process converges rapidly. The parameter changes become stable by the tenth iteration.

**Fig 18:**
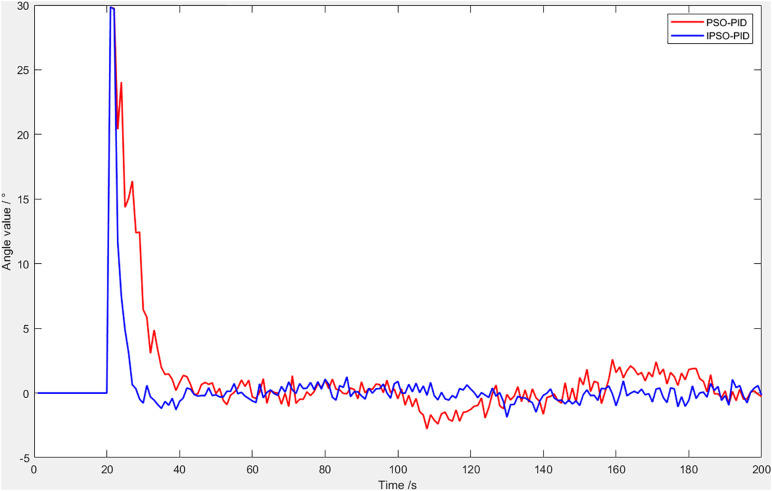
Control error comparison.

Subsequently, a control test was carried out on a single leg. The soft robotic leg was integrated with a bending sensor, which was connected to the host computer via a data acquisition card.A set of target angles was assigned within the control program, and the leg was driven to track these targets using the control algorithm.The collected data were stored in a database, then exported and used to generate plots. The resulting error comparison curve is shown in Fig 18.

The system controlled by the IPSO-PID algorithm demonstrated smaller errors between the output and the setpoint compared to the PSO-PID-controlled system. This indicates that the IPSO-PID-controlled system is more suitable for quadruped robot locomotion.

## 5. Experiment validation

### 5.1 Establishment of a control platform

**Fig 19:**
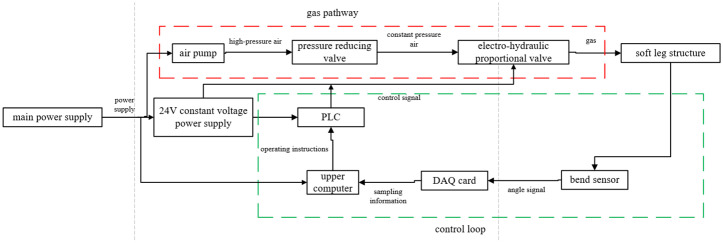
Control process flowchart.

**Fig 20:**
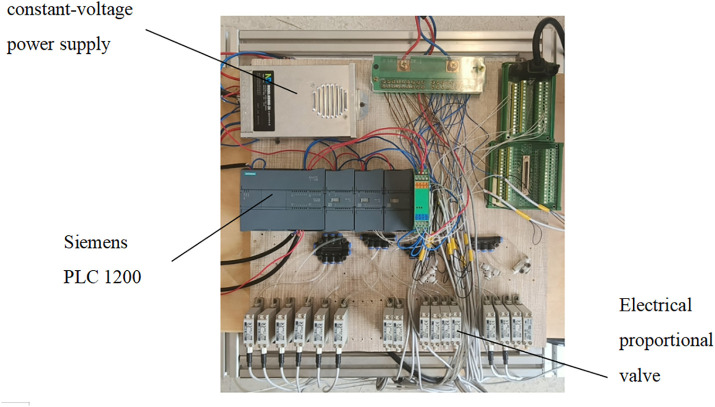
Hardware setup.proportional valve.

The control platform is utilized to drive the soft robot to achieve forward walking. This platform consists of multiple sections. The first section is the gas pathway section. The air pressure from the air pump’s pressure-reducing valve is delivered through pipelines to the input of the electrical valve. The output is then sent to various pneumatic actuators, adjusting the bending degree of the soft actuators. Secondly, the control equipment comprises the host computer, the data acquisition card, bending sensors, and the PLC. The bending sensors are affixed to the soft legs to record bending deformation data. This data is transmitted to the host computer via the data acquisition card, where the control program processes the signal and generates the output signal based on the pre-set bending angle. The PLC controller receives this signal and applies it to the electrical proportional valve, altering the air pressure. This adjustment modifies the bending state of the soft actuator to achieve the desired bending angle, completing the robot’s closed-loop control. Lastly, the power module supplies electricity to the air pump, PLC, and electrical valves. Fig 19 illustrates the flowchart of the control process, where the red-boxed section represents the gas pathway system, and the green-boxed section represents the control loop. The corresponding hardware setup isshown in Fig 20.

### 5.2 Parameter identification for the soft leg control model

To facilitate the test and identification of the soft robotic leg’s model, the initial step involves setting up the necessary hardware. This setup includes configuring signal transmission for the PLC, managing the control of the electrical proportional valves, and collecting and recording data from the bending sensors. The Access database is employed to store the collected data, while data exchange with the PLC is conducted through function modules such as TCP connections. In the program, multiple sets of data are collected, and the average value is used as the correction signal for the sensors, facilitating sensor zeroing.

**Fig 21:**
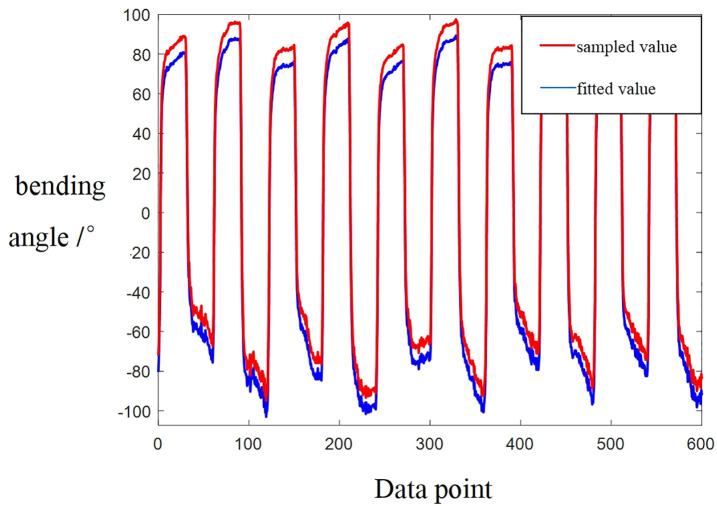
The sampled values and the fitted values.


Once the program is established and the bending sensor is calibrated, a set of input data points is selected for testing the soft robotic leg structural model. The input signals are set in a cyclic manner, with values of 1.5V, −1.5V, 2.5V, and −2.5V. When the input signal is positive, the soft robotic leg bends in the clockwise direction, whereas it bends in the counterclockwise direction for negative input values. The sampling rate is configured at 50 Hz, with 60 data points recorded at each input value, and the procedure is repeated for five complete cycles, resulting in a total of 1200 returned signals. This comprehensive dataset contains both the input and output signals, which are essential for model testing and validation. The large amount of collected data is then filtered, resampled, or transformed. The nonlinear model is applied for optimal fitting. Eventually, the parameters of the model are identified. The correspondence between the sampled values and the fitted values is shown in Fig 21 The data fitting accuracy of 94.89%, demonstrating a good fit of the model. Through a large number of repeated experiments, the transfer function of the control model is obtained as:

$$G\left(s\right)=\frac{-11.27s^{2}+34.2s+2.843}{s^{3}+1.834s^{2}+1.126s+0.06395}$$ (1.18)

To verify the stability of the model, a pole-zero analysis was performed on the identified system. The results showed that all poles were located in the left half of the complex plane, indicating that the control model is stable and controllable.The transient response curve of the system demonstrated a short rise time, fast response, and rapid convergence to steady state, further validating the feasibility, stability, and rapid responsiveness of the proposed soft leg control model.

### 5.3 Walking Experiment of a Quadruped Soft Robot Based on IPSO-PID

**Fig 22:**
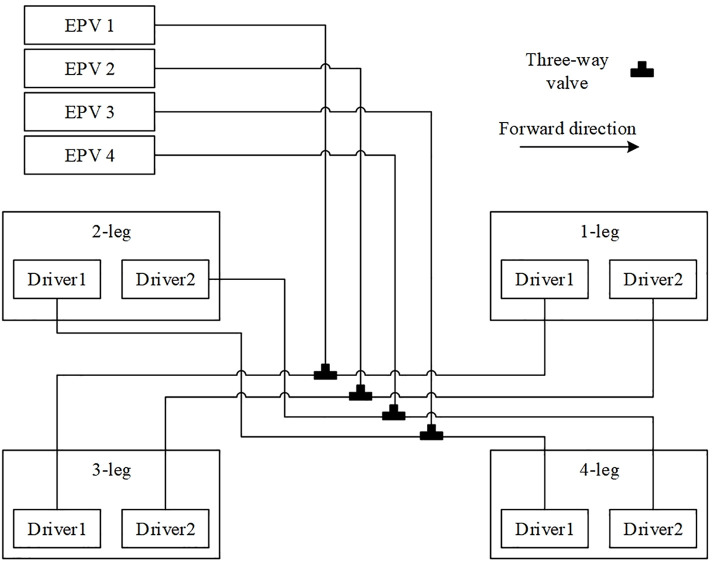
Pneumatic circuit diagram of the quadruped robot.

The pneumatic circuit diagram of the quadruped soft robot is shown in Fig 22. The robot adopts a diagonal gait for locomotion, in which the two legs on each diagonal share the same inflation logic. Therefore, the pneumatic circuits of the diagonally opposite legs are connected in parallel.

**Fig 23:**
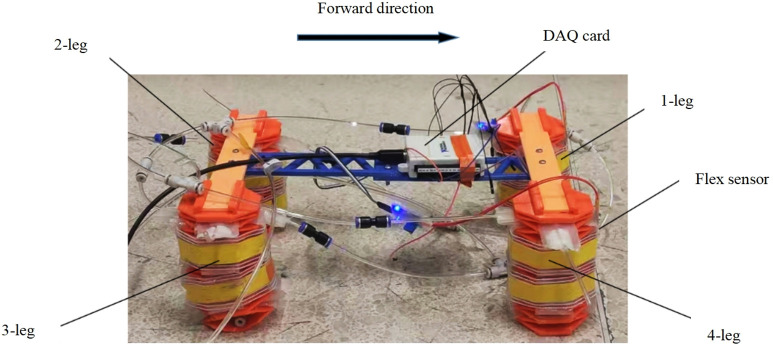
Schematic diagram of the quadruped soft robot.

As shown in Fig 23, with the arrow indicating the forward direction, the legs of the robot are labeled as Leg 1, Leg 2, Leg 3, and Leg 4. Each leg contains two internal pneumatic actuators, named Actuator 1 and Actuator 2.For each pair of diagonally opposite legs, their corresponding internal actuators are connected via pneumatic T-junctions. Each connected pneumatic loop is supplied by a single electro-pneumatic proportional valve, resulting in a total of four proportional valves used in the system.

**Fig 24:**
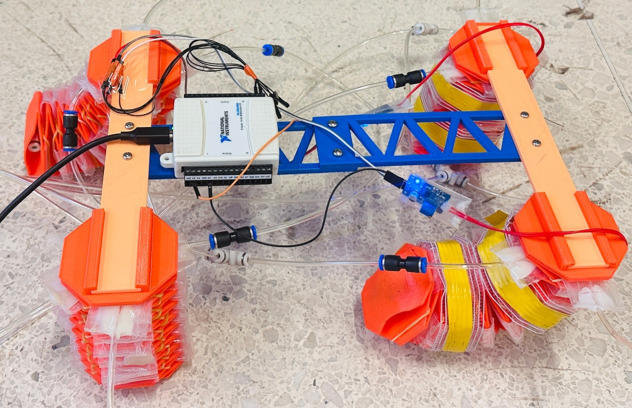
Schematic illustration of the robot’s walking gait.

The quadruped robot presented in this study adopts a diagonal gait, in which Legs 1 and 3 move synchronously, as do Legs 2 and 4.First, the front air pouches of Legs 1 and 3 contract while the rear pouches expand, causing these legs to swing forward in a stepping motion.Next, Legs 2 and 4 are controlled to bend backward while Legs 1 and 3 return to their original position, as illustrated in Fig 24. Finally, Legs 2 and 4 step forward, completing one cycle of alternating gait and achieving continuous forward locomotion.

**Fig 25:**
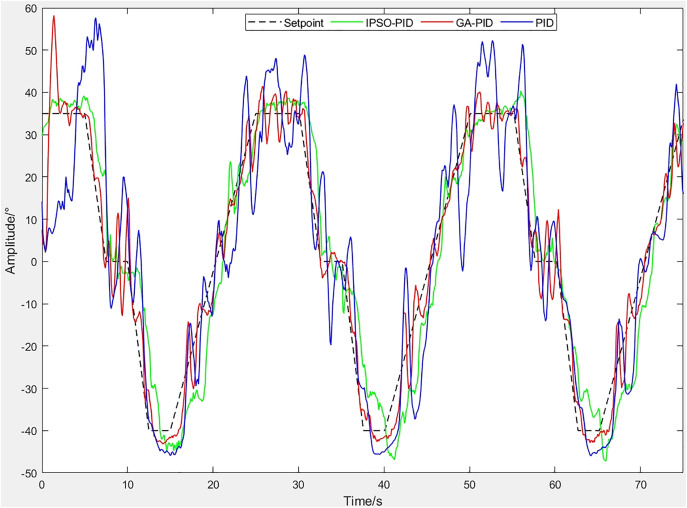
Control curves of Leg 1 and Leg 3.

**Fig 26:**
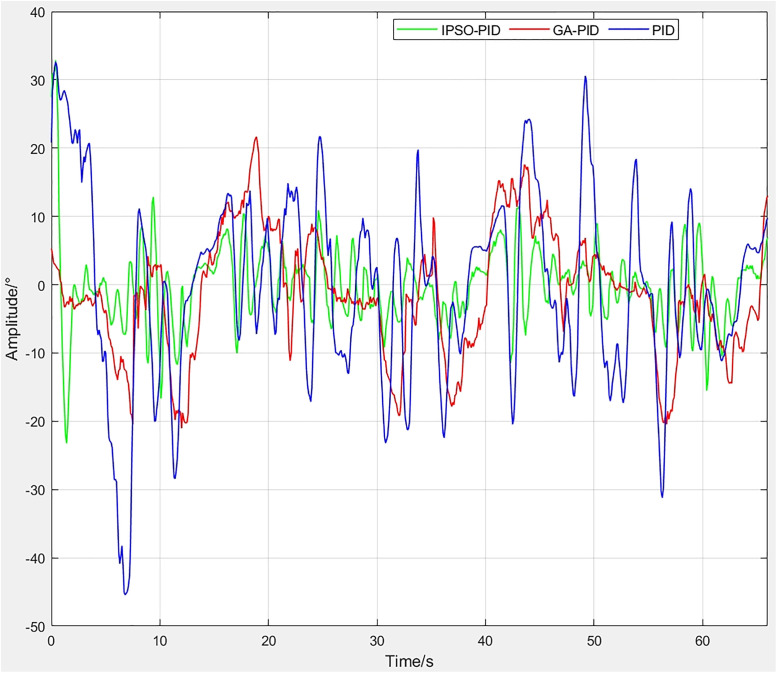
Error curves of Leg 1 and Leg 3.

The motion trajectory of the robot during stepping was recorded using an oscilloscope. Fig 25 shows the stepping control curve for the left diagonal side, while Fig 26 presents the stepping error curve for the same side.By comparing the error curves, it can be observed that the PID controller exhibits the largest fluctuations, with errors exceeding ±40° at maximum. The GA-PID achieves smaller overall errors than PID but still shows considerable oscillations. In contrast, the IPSO-PID demonstrates the smallest fluctuations, maintaining errors within ±10° for most of the time, and performs in a more stable manner. From the tracking curves, the PID controller responds quickly but produces large overshoot and fails to closely follow the reference signal. The GA-PID can follow the target trajectory, yet overshoot and lag remain around the peaks. The IPSO-PID achieves the best stability and tracking performance. Therefore, IPSO-PID is the most suitable for this nonlinear and highly compliant system.

## Conclusion

This paper presents a novel biomimetic quadruped soft robot. This paper proposed a data-driven approach for establishing the soft control model, along with an Improved Particle Swarm Optimization algorithm for tuning controller parameters.The research covers the design and optimization of the robot’s leg structure and soft actuators, the establishment of the soft control model, and the construction and experimental validation of the soft robot system. The main research content is summarized as follows:

(1) Design of Pneumatic Actuators: the pneumatic actuator suitable for a hexagonal mesh structure was designed. The pneumatic actuators achieve deformation through inflation, allowing for elongation and bending motions, thereby enabling the quadruped robot to perform stepping and walking.
(2) Modeling and Parameter Identification of Soft Actuators: the data-driven modeling approach was applied to the soft actuators control model, and the Improved Particle Swarm Optimization algorithm was proposed for automatically tuning the PID controller parameters.
(3) Construction of the Control Platform and Experimental Validation: the control platform was built, and the diagonal gait walking experiment of the quadruped soft robot was successfully conducted. Experimental results demonstrated that the proposed IPSO-PID control method reduced control errors by over 50%, proving its excellent performance in terms of control accuracy and stability.

## Supporting information

S1 Table Inputs and outputs. (XLSX)

S2 Table Bending sensor. (XLSX)

S3 Table Pressure sensor. (XLSX)

S4 Table Data of leg-1. (XLSX)

S5 Table Data of leg-2. (XLSX)

S6 Simulation Leg-1. (FIG)

S7 Simulation Leg-2. (FIG)

S8 Simulation The error of leg-1. (FIG)

S9 Simulation The error of leg-2. (FIG)
